# Variability in Susceptibility to Anthracnose in the World Collection of Olive Cultivars of Cordoba (Spain)

**DOI:** 10.3389/fpls.2017.01892

**Published:** 2017-11-06

**Authors:** Juan Moral, Carlos J. Xaviér, José R. Viruega, Luis F. Roca, Juan Caballero, Antonio Trapero

**Affiliations:** ^1^Departamento de Agronomía, Universidad de Córdoba, Córdoba, Spain; ^2^Department of Plant Pathology, Kearney Agricultural Research and Extension Center, University of California, Davis, Davis, CA, United States; ^3^Departamento de Agronomía, ETSIAM, Universidad de Córdoba, Córdoba, Spain; ^4^Departamento de Olivicultura, IFAPA Centro Alameda del Obispo, Córdoba, Spain

**Keywords:** olive, diseases, anthracnose, *Colletotrichum*, fruit rot

## Abstract

Anthracnose of olive (*Olea europaea* ssp. *europaea* L.), caused by *Colletotrichum* species, is a serious disease causing fruit rot and branch dieback, whose epidemics are highly dependent on cultivar susceptibility and environmental conditions. Over a period of 10 years, there have been three severe epidemics in Andalusia (southern Spain) that allowed us to complete the assessment of the World Olive Germplasm Bank of Córdoba, one of the most important cultivar collections worldwide.A total of 308 cultivars from 21 countries were evaluated, mainly Spain (174 cvs.), Syria (29 cvs.), Italy (20 cvs.), Turkey (15 cvs.), and Greece (16 cvs.). Disease assessments were performed using a 0–10 rating scale, specifically developed to estimate the incidence of symptomatic fruit in the tree canopy. Also, the susceptibility of five reference cultivars was confirmed by artificial inoculation. Because of the direct relationship between the maturity of the fruit and their susceptibility to the pathogen, evaluations were performed at the end of fruit ripening, which forced coupling assessments according to the maturity state of the trees. By applying the cluster analysis to the 308 cultivars, these were classified as follows: 66 cvs. highly susceptible (21.4%), 83 cvs. susceptible (26.9%), 66 cvs. moderately susceptible (21.4%), 61 cvs. resistant (19.8%), and 32 cvs. highly resistant (10.4%). Representative cultivars of these five categories are “Ocal,” “Lechín de Sevilla,” “Arbequina,” “Picual,” and “Frantoio,” respectively. With some exceptions, such as cvs. Arbosana, Empeltre and Picual, most of the Spanish cultivars, such as “Arbequina,” “Cornicabra,” “Hojiblanca,” “Manzanilla de Sevilla,” “Morisca,” “Picudo,” “Farga,” and “Verdial de Huévar” are included in the categories of moderately susceptible, susceptible or highly susceptible. The phenotypic evaluation of anthracnose reaction is a limiting factor for the selection of olive cultivars by farmers, technicians, and breeders.

## Introduction

Olive (*Olea europaea* ssp. *europaea* L.) is the most extensively planted fruit crop in the world, covering more than 10.2 million hectares of land, mainly in the Mediterranean Basin. Almost 25% of the total olive trees are grown in Spain, where more than 45% of the world's olive oil is produced (FAO, 2015). Olive industry (oil and table) is a vital sector of Spanish agro-food system with a total value of 3 billion euros, more than 385,000 agricultural holdings, and over half million farmers (INE, 2015)^1^ In fact, the global price of olive oil yearly depends to a large extent to the Spanish production.

Olive oil has numerous beneficial properties, mainly associated with its high content of monounsaturated oleic acid (Espósito et al., 2004) albeit other minor components, such as phenolic compounds (i.e., hydroxytyrosol, oleocanthal, and squalene) have also shown substantial benefits for consumer health (Beauchamp et al., 2005). For this reason, olive oil consumption and, concomitantly the olive-growing area, have notably increased worldwide in recent years. Projects to expand the olive-growing surface affect new areas for this crop, such as different provinces of China, India, Saudi Arabia, or the States of Florida and Hawaii in the USA. In many of these plantation areas, the adaptation of the different olive cultivars and the pests and diseases of this crop are unknown. Fortunately, the technicians and farmers have a broad range of cultivars that can be selected according to their adaptation to the target agro-environment area.

Olive tree was probably domesticated over 6,000 years ago in the Middle East, from which it spread until covering the entire Mediterranean Basin. It is also accepted the existence of other diversification centers across Mediterranean Basin (Besnard et al., 2013; Díez et al., 2015). The first farmers selected the most outstanding individuals in each olive-growing area according to their adaptation to the soil and climate and their agronomic characteristics. These original cultivars were subsequently maintained by vegetative propagation and, in general, have remained confined to small areas (Rallo et al., 2005; Díez et al., 2015). The presence of homonymy (different cultivars with the same name in different zones), synonymy (a given cultivar with various names in the areas that it occupies), and wrong denominations is frequent in this crop (Ganino et al., 2006; Trujillo et al., 2013). Thereafter, the exact number of olive cultivars is unknown but it is likely that this number reaches around 2000 (Bartolini et al., 1998). For this reason, and due to the absence of systematic studies, there is a reduced classification of the olive cultivars according to their susceptibility to many diseases, particularly in the case of anthracnose (Moral and Trapero, 2009).

Olive anthracnose, caused by the fungal complex species *Colletotrichum acutatum sensu lato* (*s. lat*.), *C. boninense s. lat*., and *C. gloeosporioides s. lat*. is the most destructive disease of olive fruit and is widely distributed in many olive-growing regions of the world (Martín and García-Figueres, 1999; Talhinhas et al., 2005; Cacciola et al., 2012; Moral et al., 2014; Schena et al., 2014). About 13 *Colletotrichum* species, belonging to these three complex species have been described affecting this crop (Talhinhas et al., 2005; Schena et al., 2014; Chattaoui et al., 2016). In general, several *Colletotrichum* species coexist in each olive-growing region with one or two dominant species and several secondary (Faedda et al., 2011; Moral et al., 2014). For example, the species *C. godetiae* (syn. *C. clavatum*) and *C. acutatum sensu stricto* (*s. str*.) are dominant in olive orchards of southern Italy (Schena et al., 2017); while *C. acutatum s. str*. and *C. gloeosporioides s. s*. are dominant and secondary species, respectively, in Tunisia (Chattaoui et al., 2016).

Olive anthracnose is also called soapy fruit due to its characteristic fruit-rot syndrome with a profuse production of spores in a gelatinous matrix under wet conditions (Moral et al., 2009). In addition to the direct losses due to the premature fall of affected fruit, phytotoxins produced by the pathogen in the rotten fruit cause a second syndrome, the dieback of shoots and branches (Ballio et al., 1969; Moral et al., 2009). Furthermore, even with low fruit-rot incidence (5%), the olive oil coming from affected orchards shows poor chemical and organoleptic characteristics that restrict or impede its commercialization as extra virgin olive oil (Moral et al., 2014).

Both cultivar susceptibility and weather conditions profoundly influence on the olive anthracnose severity (Moral and Trapero, 2012). For example, in the central provinces of Andalusia, where the susceptible cultivars Hojiblanca and Picudo grow, severe epidemics occur if the weather conditions are conducive during the autumn despite the farmers typically applying a 2–3 copper-based fungicide treatments during this period. In southern Portugal, where autumn-winter is more humid than in central Andalusia, severe epidemics occur in super-high-density (hedgerow system) olive orchards planted with the moderately susceptible cultivar Arbequina (Moral and Trapero, 2012; Moral et al., 2014).

Although satisfactory results controlling olive anthracnose can be obtained using inorganic and organic fungicides, field application is not always effective for different reasons, such as: (i) the number of registered fungicides in post-bloom is very small; (ii) *Colletotrichum* shows a high tolerance to copper, the basic ingredient of the main fungicides used; (iii) and the optimum period for fungicide application is relatively short (Roca et al., 2007; Cacciola et al., 2012; Moral et al., 2014). Since the unripe fruit are resistant to the pathogen, the use of early harvesting before the fruit reaches full ripening or the selection of late-maturing cultivars are efficient and environmentally friendly control measures (Moral et al., 2008). However, these practices have some agronomical inconveniences: (i) if the fruit is immature (not completely black), it usually shows less oil content than mature fruit; (ii) the immature fruit show a high fruit retention force being difficult its mechanical harvest; and (iii) when the fruit still immature (green) during the winter, it is highly sensitive to frost damage (Rallo et al., 2005). Therefore, the use of resistant olive cultivars to anthracnose is the most effective control method, which does not show the previous inconvenience, and can be combined with other measures, such as biological and chemical methods or cultural practices (Moral et al., 2008; Moral and Trapero, 2009; Preto et al., 2017).

The World Olive Germplasm Bank of Córdoba (WOGBC) from Andalusia region, southern Spain, is a magnificent setting to evaluate the susceptibility of the olive cultivars to aerial diseases, including anthracnose, for several reasons: (i) the WOGBC is currently on of the largest olive germplasm banks with more than 900 accessions and 411 cultivars from 24 countries (A. Belaj, unpublished data); (ii) it is located in an endemic anthracnose area (Moral et al., 2015); and (iii) the whole group of olive trees of WOGBC has been identified using Simple Sequence Repeats (SSR) markers, resolving the discrepancies due to misidentification of the trees (Trujillo et al., 2013). During the last 10 years, we have developed and validated laboratory and field methods to evaluate the susceptibility of olive cultivars to anthracnose (Moral et al., 2008; Moral and Trapero, 2009). These methods have been used to assess the resistance of traditional olive cultivars and new ones, coming from the breeding program of the University of Córdoba (UCO) and the Andalusian Institute for Research and Formation in Agriculture and Fishery (IFAPA) (Moral and Trapero, 2009; Moral et al., 2015). While the disease reaction of some olive cultivars is well-known (Moral and Trapero, 2009; Talhinhas et al., 2015), most of cultivars are still unclassified for their resistance to this pathogen (Moral et al., 2014).

Because the olive cultivars from WOGBC have not been systematically screened for resistance to *Colletotrichum*, the objectives of this study were the following: (i) to assess the susceptibility of cultivars in the WOGBC to anthracnose caused by *Colletotrichum* spp.; (ii) to identify cultivars representative of each of the susceptibility categories determined in this study; and (iii) to correlate the anthracnose susceptibility with other phenotypic characteristics of cultivars. The phenotypic evaluation of disease reaction is a limiting factor for the selection of olive cultivars by farmers, technicians, and breeders.

## Materials and method

### Plant material and orchard

We have evaluated 308 accessions of cultivated olive trees from 22 countries of origin (Table 1). These accessions are conserved as a live collection in the WOGBC located in a 5.2-ha flat and uniform field (37.51°N, 4.18°W, altitude 113 m) at the IFAPA, Center “Alameda del Obispo.” The soil of the orchard was classified as a Typic Xerofluvent with a sandy-loam texture, and the climatic conditions were typical of the Mediterranean area (García-López et al., 2016). The experimental orchard is located ≈1 Km from the main river of Andalucía, Guadalquivir River, in a humid area where anthracnose is an endemic disease (Moral et al., 2015). Because these accessions are fully authenticated by Trujillo et al. (2013), we will refer to them as cultivars. The WOGBC currently contains more than 411 cultivars (Caballero et al., 2006; A. Belaj, unpublished data). Olive trees used were planted between 1982 and 1992, so they were at least 5-years-old trees when evaluated. Because the experimental plot has a completely randomized design, several anthracnose susceptible cultivars (Table 1) are randomly distributed in the WOGBG resulting in a homogeneous distribution of *Colletotrichum s. lat*. inoculum, as with other pathogens, such as *Venturia oleaginea* (López-Doncel, 2003; Moral, 2009). In this study, which covers the period 1997–2008, we present the result of the evaluation of 308 well-identified cultivars according to their reaction to the pathogen. The remaining cultivars were not evaluated for different reasons: tree loss by *Verticillium dahliae*, absence of yield, absence of replicated trees of the same cultivar, etcetera. Each of the 308 evaluated cultivars has from two to 22 replicated trees randomly distributed in the experimental plot.

**Table 1:** Reaction of olive cultivars to anthracnose, caused by *Colletotrichum acutatum*, in the World Olive Germplasm Bank of Córdoba (Spain).

**Reaction**	**No**	**Cultivar^a^**
Highly Susceptible (HS)	66	Abbadi, Acebuchera, Adkam, Agouromanakolia, Azapa, Bolvino, Borriolenca, Bouteillan, Cañivano Negro-55, Chalchali, Chorruo de Castro del Río, Corbella, Cornicabra de Mérida, Dulzal de Carmona, Farga, Forastera de Tortosa, Fulla de Salze, Gatuno, Gerboui, Gordal Sevillana, Grosal de Cieza, Habichuelero de Baena, Hojiblanca, Imperial, Imperial de Jaén, Jabaluna, Jaropo, Limoncillo, Llorón de Atarfe, Loaime, Lucio, Lucques, Machorrón, Mahati-1010, Manzanilla de Almería, Manzanillera de Huércal, Manzanillo de Cabra, Merhavia, Meski, Morisca, Morisca de Mancor, Negrillo de Iznalloz, Negrillo de la Carlota, Negrillo Redondo, Nevadillo de Santisteban del Puerto, Nevado Azul, **Ocal**, Ocal-25, Ojo de Liebre, Palomar, Pavo, Picudo, Picudo Blanco de Estepa, Rachati, Rechino, Safrawi, Salgar Redondo, Salonenque, Sant Agostino, Sayfi, Sevillana de Abla, Sevillenca, Temprano, Uovo di Piccione, Uslu, and Verdial de Cádiz.
Susceptible (S)	83	Abbadi Abou Gabra-84, Abbadi Shalal, Abou Kanani, Adramitini, Alameño Blanco, Alameño de Cabra, Alameño de Montilla, Amargoso, Amygdalolia Nana, Argudell, Ascolana Tenera, Asnal, Ayvalik, Barri, Blanqueta, Carolea, Castellana, Chorreao de Montefrío, Cipressino, Cirujal, Cordobés de Arroyo de la luz, Cordovil de Castelo Branco, Cornezuelo de Jaén, Cornicabra, Doebli, Dulzal, Escarabajillo, Escarabajuelo de Atarfe, Escarabajuelo de Posadas, Figueretes, Galega Vulgar, Gemlik, Gordal de Granada, Gordal de Hellín, Gordal de Vélez Rubio, Grosal Vimbodí, Izmir Sofralik, Jlot-841, Kaesi, Kalamon, Kolybada, **Lech**í**n de Sevilla**, Lentisca, Levantinka, Macho de Jaén, Mahati-846, Manzanilla de Piedra Buena, Manzanilla de Agua, Manzanilla de Sevilla, Manzanilla Prieta, Mastoidis, Mawi, Mollar Basto, Mollar de Cieza, Morona Negral de Sabiñan, Negrillo de Estepa, Negro del Carpio, Nevadillo Blanco de Jaén, Nevado Basto, Nevado Rizado, Olivo de Mancha Real, Pajarero, Perafort, Pico Limón de Grazalema, Picual de Almería, Polinizador, Racimal, Shami, Sorani, Tempranillo de Yeste, Torcio de Huelma, Toruno, Varudo, Vera, Verdal de Manresa, Verdala, Verdale, Verde Verdelho, Verdial de Badajoz, Verdial de Huévar, Verdiell, and Zarza.
Moderately Susceptible (MS)	66	Abou Satl Mohazam, **Arbequina**, Arroniz, Azulejo, Barnea, Beyaz Yaglik, Bodoquera, Caballo, Cañivano Negro, Carrasquenho de Elvas, Carrasqueño de Alcaudete, Carrasqueño de la Sierra, Carrasquillo, Cerezuela, Changlot Real, Chorruo, Çobrancosa, Cordovil de Serpa, Cornicabra de Jerez, Cornicabra-1, Domat, Dwarf D, Erbek Yaglik, Escarabajuelo de Úbeda, Gaydoyrelia, Genovesa, Habichuelero de Grazalema, Itrana, Kelb et Ter, Khashabi, Kiraz, Klon-14-1081-1, Klon-14-1081-2, Konservolia, Lastovka, Lechín de Granada, Maarri, Majhol-1059, Manzanilla Cacereña, Manzanilla de Abla, Manzanilla del Piquito, Mari, Masabi, Mission, Moojeski, Morrut, Myrtolia, Negrillo de Arjona, Oblica, *Olea ferruginea*, Olivo de Maura, Picholine, Plementa Bjelica, Pulazeqin, Rapasayo, Redondilla de Logroño, Reixonenca, Royal de Calatayud, Royal de Cazorla, Royal de Sabiñan, Sabatera, Sandalio, Tanche, Toffahi, Valanolia, Villalonga, and Vinyols.
Resistant (R)	61	Abbadi Abou Gabra-10, Acebuche de Caravaca, Alfafara, Aloreña de Iznalloz, Arbosana, Athalassa, Beladi, Belluti, Bent al Kadi, Biancolilla, Bical, Buga, Buidiego, Canetera, Carrasqueño de Jumilla, Carrasqueño de Porcuna, Chalkidikis, Chemlal de Kabilye, Chemlali, Chetoui, Coratina, Corralones de Andújar, Curivell, Datilero, Dokkar, Dolce, Elmacik, Enagua de Arenas, Fishomi, Grit Eytini, Grossanne-67, Joanenca, Kalokerida, Kotruvsi, Leccino, Llumeta, Majhol-1013, Majhol-152, Manzanilla de Hellín, Manzanilla de Montefrío, Manzanilla de San Vicente, Maurino, Memecik, Mixani, Moraiolo, Patronet, Pecoso, Picholine Marocaine, Pico Limón, **Picual**, Real Sevillana, Redondilla de Grazalema, Sevillano de Jumilla, Shengue, Sinop, Sollana, Vallesa, Vaneta, Verdial de Vélez-Málaga, Zaity, and Zard.
Highly Resistant (HR)	32	Ayrouni, Azul, Bosana, Callosina, Caninese, Crnica, Dolce Agogia, Empeltre, **Frantoio**, Frantoio A. Corsini, Grappolo, Istarska Bjelica, Kan Çelebi, Kato Drys, Koroneiki, Mavreya, Megaritiki, Menya, Ouslati, Pendolino, Pequeña de Casas Ibañez, Perillo de Jaén, Piñonera-76, Racimal de Jaén, Rosciola, Royal de Calatayud-4, Selvatico, Toffahi-1000, Ulliri i Bardhe i Ti, Ulliri i Kuq, Wardan, and Zalmati.

a *Reference cultivars appear in bold*.

The olive trees were planted in a 7 × 7 m square using one trunk per plant. The trees were initially pruned to select three or four main branches to form the canopy structure: a free open vase. After that, the olive trees were periodically pruned to renewal branches or to eliminate dead branches. The experimental orchard was irrigated during spring-summer applying over 2,000 m^3^ of water per year using drip irrigation. Three Bordeaux mixture treatments (Caldo Bordelés Vallés, IQV, 2 kg active Cu++ per ha and treatment) were applied throughout the year at the end of winter (February–March), spring (April–May), and autumn (October) (Roca et al., 2007). According to olive pest, it should be noted that the population of the olive fruit fly (*Bactrocera oleae*) is well established in the WOGBG area albeit there are not specific studies about it (Caballero J., unpublished data).

In previous studies, we identified the population of the pathogen in the WOGBC as *C. acutatum* group A4 (Moral et al., 2015), which is the only group described in the central provinces of Andalusia (Moral et al., 2014). The molecular group A4 was reclassified as *C. clavatum species nova* (sp. nov.) by Faedda et al. (2011). However, Damm et al. (2012) showed then that *C. clavatum* is synonym of the previous species *C. godetiae*. Because the latter name is rarely used in non-etiology studies, we maintain the generic name *C. acutatum s. lat*. throughout the present article.

### Assessment of disease incidence in the field

Fruit-rot incidence was assessed in each olive tree using a previously described and validated 0–10 rating scale (Moral and Trapero, 2009). The rating scale is the logistic transformation of the proportion of symptomatic fruit and it based on the sigmoidal equation:

(1) $$\begin{array}{l} Y=\frac{100}{1+3^{\left(7-X\right)}} \end{array}$$

in which *Y* = percentage of affected fruit and *X* = scale value (0–10). The fruit-rot incidence data are normalized using this scale, and the scale values can be analyzed directly using parametric methods (Moral and Trapero, 2009; Table 2). When the olive trees showed a high percentage (>5%) of symptomatic fruit, the assessor directly quantified the percentage of symptomatic fruit on the tree canopy. The percentage of affected fruit was then transformed in scaling rating values (0–10) rewriting the above equation as:

(2) $$\begin{array}{l} X=7-\frac{Ln\left(\frac{100}{Y}-1\right)}{Ln3}=7-Log_{3}\left(\frac{100}{Y}-1\right) \end{array}$$

**Table 2:** Rating scale values, average, and interval of percentage of olive fruit affected by anthracnose.

**Scale value (0–10)**	**Description**	**Average (Interval [%]^a^)**	**Range^b^**
0	Affected fruit not observed	<0.04	0.04
1	From one to three affected fruit per olive tree	0.14 (0.04^c^-0.23)	0.19
2	From one to three affected fruit per each quadrant of the tree canopy	0.41 (0.24–0.70)	0.46
3	From four to nine affected fruit per each quadrant of the tree canopy	1.22 (0.71–2.09)	1.38
4	Affected fruit easily detected (from 20 to 36 affected fruit per each half of the tree canopy)	3.57 (2.10–6.02)	3.92
5	Direct quantification of affected fruit	10 (6.03–16.13)	10.1
6	Direct quantification of affected fruit	25 (16.14–36.60)	20.46
7	Direct quantification of affected fruit	50 (36.61–63.39)	26.78
8	Direct quantification of affected fruit	75 (63.40–83.86)	20.46
9	Direct quantification of affected fruit	90 (83.87–93.97)	10.1
10	<36 asymptomatic fruit per each half of the tree canopy	97 (93.98–100)	3.92

a *Values taken from the logistic function $Y=\frac{100}{1+3^{\left(7-X\right)}}$in which Y = percentage of affected fruit and X = scale value*.

b *Range of affected fruit (%) for each scale value*.

c *Detection limit of visual assessments in the field (one affected fruit from 2,500 observed fruit per tree; Moral and Trapero, 2009)*.

Disease incidence was assessed at different times when most the fruit showed a value 4 in the 0 to 4 rating scale (from green to black, respectively) for olive fruit ripening (Rallo et al., 2005). Disease incidence was assessed from mid-December to the end of January in 1997–1998, 2005–2006, and 2006–2007 when severe epidemics of olive anthracnose occurred in the experimental orchard (Moral and Trapero, 2009). Data from low-level epidemic years were not considered because these data only divide the olive cultivars into two groups, the highly susceptible cultivars, which show a low-medium incidence of fruit rot, and the rest of cultivars showing no symptomatic fruit (Moral and Trapero, 2009; Xaviér, 2009).

Because at the beginning of the spring 2007 there was an important peak of the dieback syndrome (chlorosis and wilting of leaves and dieback of shoots and branches, i.e., green and lignified shoots, respectively) after two epidemic years, we assessed this second syndrome according to volume of tree canopy affected using the following rating scale: 0 < 10%, 1 = 10–24%, 2 = 25–49%, 3 = 50–74%, 4 = 75–90%, and 5 ≥ 90% (Moral and Trapero, 2009). In addition, we evaluated the presence of the pathogen on symptomatic tissues (leaves and dieback shoots) by culturing small pieces on acidified Potato Dextrose Agar plus 100 mg of copper sulfate (CuSO_4_·5H_2_O) per liter (Moral et al., 2009) from May to August during 2006 and 2007.

### Inoculation of detached fruit

Apparently asymptomatic yellowish-green—value of 2 on the olive ripening scale (Rallo et al., 2005)—fruit of five cultivars (“Arbequina,” “Frantoio,” “Lechín de Sevilla,” “Ocal,” and “Picual”) were collected at the onset of ripening from olive trees in the experimental orchard during the non-epidemic year 2012. These reference cultivars were selected by their well-known response to anthracnose in the field (Moral et al., 2014, 2015). The fruit were inoculated and incubated according to Moral et al. (2008). Briefly, fruit were washed, disinfested, and sprayed with a conidial suspension (10^5^ conidia per ml or sterile water for the control) of isolate Col-104 of *C. acutatum s. lat*. Inoculated and control fruit were incubated in humid chambers (plastic containers) at 22–24°C under fluorescent lights (12 h alternating photoperiod, 40 μmol m^−2^s^−1^). Disease severity was periodically assessed for 80 days using a 0–5 rating scale where 0 = no visible symptoms, 1 = visible symptoms affecting <25% of the fruit surface, 2 = 25–49%, 3 = 50–74%, 4 = 75–100%, and 5 = soapy fruit (Moral et al., 2008). There were two replicates (moist chambers) per treatment and 25 fruit per replicate arranged in a completely randomized design. The experiment was repeated once and data analyses were performed on the pooled data from the two replicates.

### Statistical analysis

To estimate the phenotypic stability of the olive cultivar on anthracnose reaction during the three studied seasons, we calculated the proportion of variability explained by the cultivar, season, and the interaction cultivar-season. For that, analysis of variance (ANOVA) was performed on the rating scale data of fruit-rot incidence of the cultivars, which were evaluated during the three epidemic seasons with a representative number of repetitions (at least two olive trees of the same cultivar). Subsequently, we calculated eta squared (η^2^) as the ratio the variability associated with an effect and the total variability of our analysis $\left(\eta^{2}=\frac{SS_{effect}}{SS_{Total}}\right)$. Because η^2^ overestimates the effect size, we also calculated partial omega squared (ω^2^) using the equation (Fritz et al., 2012):

(3) $$\begin{array}{l} \omega^{2}=\frac{SS_{effect}-df_{effect}\times MS_{error}}{SS_{total}+\;MS_{error}} \end{array}$$

in which *SS* = sum of squares, *df* = degrees of freedom, and *MS* = mean square. Likewise, ANOVA was performed on the rating scale data of fruit-rot incidence and branch dieback severity of the five reference cultivars.

In previous work, we have classified 18 cultivars into three categories of susceptibility by comparing their reaction according to the disease response of moderately resistant cultivar Arbequina (Moral et al., 2015). In this study, due to the high number of studied cultivars and, for establishing groups more homogeneous according to their reaction to the pathogen, the cultivars are classified roughly into five categories. These five susceptibility categories were determined according to the reaction of the reference cvs. Arbequina, Frantoio, Lechín de Sevilla, Ocal, and Picual, which were selected by their well-known response to anthracnose under field conditions (Moral et al., 2005, 2014, 2015). Subsequently, a non-hierarchical K-Means cluster analysis with the initial cluster center method under the assumption of five groups (*k* = *5*, one group for each of the reference cultivars) was applied to the classification of the whole of the cultivars using the average value of the epidemic seasons due to the fact that it is the best parameter to discriminate the susceptibility/resistance reaction of the cultivars (Moral and Trapero, 2009; Table 2). The relationship between fruit-rot incidence and branch-dieback severity were analyzed by the non-parametric Spearman rank correlation. A chi-squared test was used to determine whether the country of origin of the cultivars had any effect on the frequency of resistant or susceptible cultivars.

In the artificial inoculation test, disease severity values of inoculated fruit were used to calculate the McKinney's Index (MKI; McKinney, 1923), in which disease severity is expressed as a percentage of the maximum possible level according to the following formula:

(4) McKinney's Index=∑(ni×i)5×N× 100

where *i* represents the severity of symptoms (0–5), *n*_*i*_ is the number of fruit with the severity of *i*, and *N* is the total number of evaluated fruit. For each cultivar and replication, the standardized area under the disease progress curve (SAUDPC) was calculated by trapezoidal integration of MKI values over time (80 days) expressed as a percentage of a maximum theoretical curve. The SAUDPC was transformed to arcsin$\sqrt{SAUDPC/100}$ when necessary for homogeneity of variance. ANOVA was performed on the SAUDPC data, and treatment means were compared using LSD test at *P* = 0.05. Eta squared (η^2^) and omega squared (ω^2^) were computed in MS Excel (Microsoft, Redmond, WA). Data from all experiments were analyzed using Statistix 9 (Analytical Software, Tallahassee, FL) except K-Means Cluster Analysis that was conducted using the SPSS 16 software.

## Results

### Susceptibility of cultivars in the field

**Figure 1:**
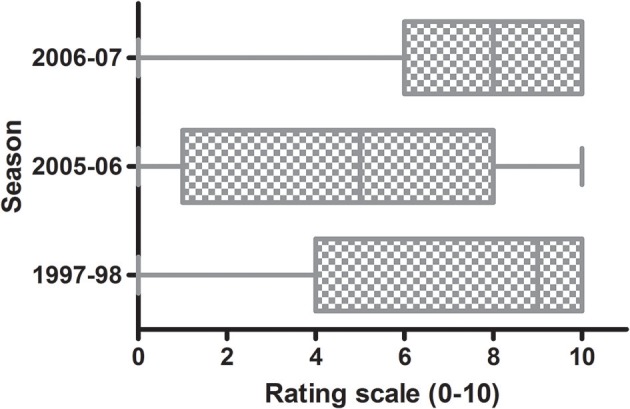
Box plots of fruit-rot incidence (0–10) caused by *Colletotrichum acutatum* in 308 olive cultivars in the World Olive Germplasm Bank of Córdoba in three epidemic seasons. Each box represents the distribution of fruit-rot incidence data according to rating scale 0–10 (Moral and Trapero, 2009). The line within the box is the median. The top and bottom lines of the box represent 25 and 75th percentile of the data. Lines extending horizontally beyond the box represent the 5 and 95th percentiles.

The incidence of fruit affected by *C. acutatum s. lat*. on the trees of the WOGBC was evaluated during three seasons (1997–1998, 2005–2006, and 2006–2007) due to the low fruit-rot incidence during the remaining years. Among these epidemic seasons, the most severe outbreak occurred in 1997–1998, whereas the lowest levels of the disease occurred in 2005–2006. For example, about 50% of the evaluated olive trees had a rating value scale ≥ 8 (i.e., median ≈8) in 1997–1998 and 2006–2007, while the media value was around 5 in 2005–2006 indicating a higher degree of dispersion (Figure 1). In the studied seasons, the fruit-rot incidence greatly varied among cultivars and season. According to η^2^ and ω^2^, the cultivar was the most important factor explaining the total variance (approximately 60%) followed by the season and the interaction genotype-season. In other words, the differences in severity of symptoms among olive trees during the epidemic seasons were mainly due to the genotype.

Because cultivar reaction to the pathogen ranged continuously from highly susceptible to highly resistant, we selected five cultivars across the susceptibility range. Each of the selected cultivars was represented by at least five repetitions (trees) in the experimental plot. These cultivars were: “Frantoio” (*n* = 8 trees) for the Highly Resistant cultivars (HR, 0–2 in the rating scale), “Picual” (*n* = 8 trees) for the Resistant cultivars (R, 2.1–4.3 in the rating scale), “Arbequina” (*n* = 6 trees) for the Moderately Susceptible cultivars (MS, 4.4–6.2 in the rating scale), “Lechín de Sevilla” (*n* = 5 trees) for the Susceptible cultivars (S, 6.3–7.9 in the rating scale), and “Ocal” (*n* = 21 trees) as representative of the Highly Susceptible cultivars (HS, > 8 on the rating scale). The fruit-rot incidence caused by *C. acutatum s. lat*. on the five selected cultivars varied significantly (*P* < 0.05) among them albeit these differences depended on the year (Table 3). Concurrent with the greatest anthracnose epidemic in 1997–1998, we detected main differences among these cultivars according to their anthracnose resistance. The cultivars Picual (R) and Arbequina (MS) did not differ significantly from each other during the three epidemic seasons according to the fruit-rot incidence, although the difference was significant when the analysis was applied to the average fruit-rot incidence of the three seasons. Likewise, the branch-dieback severity showed by reference cultivars differed significantly, being particularly severe in “Ocal” (Table 3). There was a significant and positive correlation (Spearman's correlation; *r* = 0.691, *P* < 0.0001) between the fruit-rot incidence and the volume (%) of tree canopy affected by the pathogen for these five cultivars.

**Table 3:** Incidence of anthracnose in five olive cultivars in the World Olive Germplasm Bank of Córdoba during 1997–1998, 2005–2006, and 2006–2007.

**Cultivar**	**Fruit-rot incidence (0–10)^a^**				**Branch^b^ dieback (0–5)**
	**1997–1998**	**2005–2006**	**2006–2007**	**Average**	**2007**
Frantoio	0.1d^c^	0.0d	0.1d	0.1e	0.0c
Picual	3.5c	1.0c	4.7c	3.4d	0.0c
Arbequina	4.5c	3.7bc	5.5c	4.9c	0.14b
Lechín de Sevilla	8.3b	5.3b	7.1b	7.0b	0.17b
Ocal	9.9a	7.7a	9.6a	9.2a	1.38a
Mean	6.5	5.1	6.1	5.7	0.34

a *Fruit-rot incidence was estimated using a 0–10 rating scale in which binary data (proportion of affected fruit) are normalized by applying the logit transformation of proportion. Scale values were directly subjected to analysis of variance and mean comparison tests*.

b *Volume of olive tree canopy affected dieback syndrome (chlorosis and wilting of leaves and dieback of shoots and branches) according to the following rating scale: 0 < 10%, 1 = 10–24%, 2 = 25–49%, 3 = 50–74%, 4 = 75–90%, and 5 = > 90% (Moral and Trapero, 2009)*.

c *Means with the same letter are not significantly different according to the Fisher's protected LSD test at P = 0.05*.

Due to the large number of cultivars evaluated, the results showed a continuous gradation in the susceptibility of cultivars ranging from completely resistant to extremely susceptible. In other words, the resistance ranged from cultivars with none or very few affected fruit (e.g., “Dolce Agogia,” “Frantoio,” “Grappolo,” or “Mavreya”), to cultivars with all affected fruit (e.g., “Acebuchera,” “Picudo Blanco de Estepa,” or “Uovo Piccione”). By applying the cluster analysis to the 308 olive cultivars, these were classified as follows: 32 cvs. HR (10.4%), 61 cvs. R (19.8%), 66 cvs. MS (21.4%), 83 cvs. S (26.9%), and 66 cvs. HS (21.4%) (Table 1).

**Figure 2:**
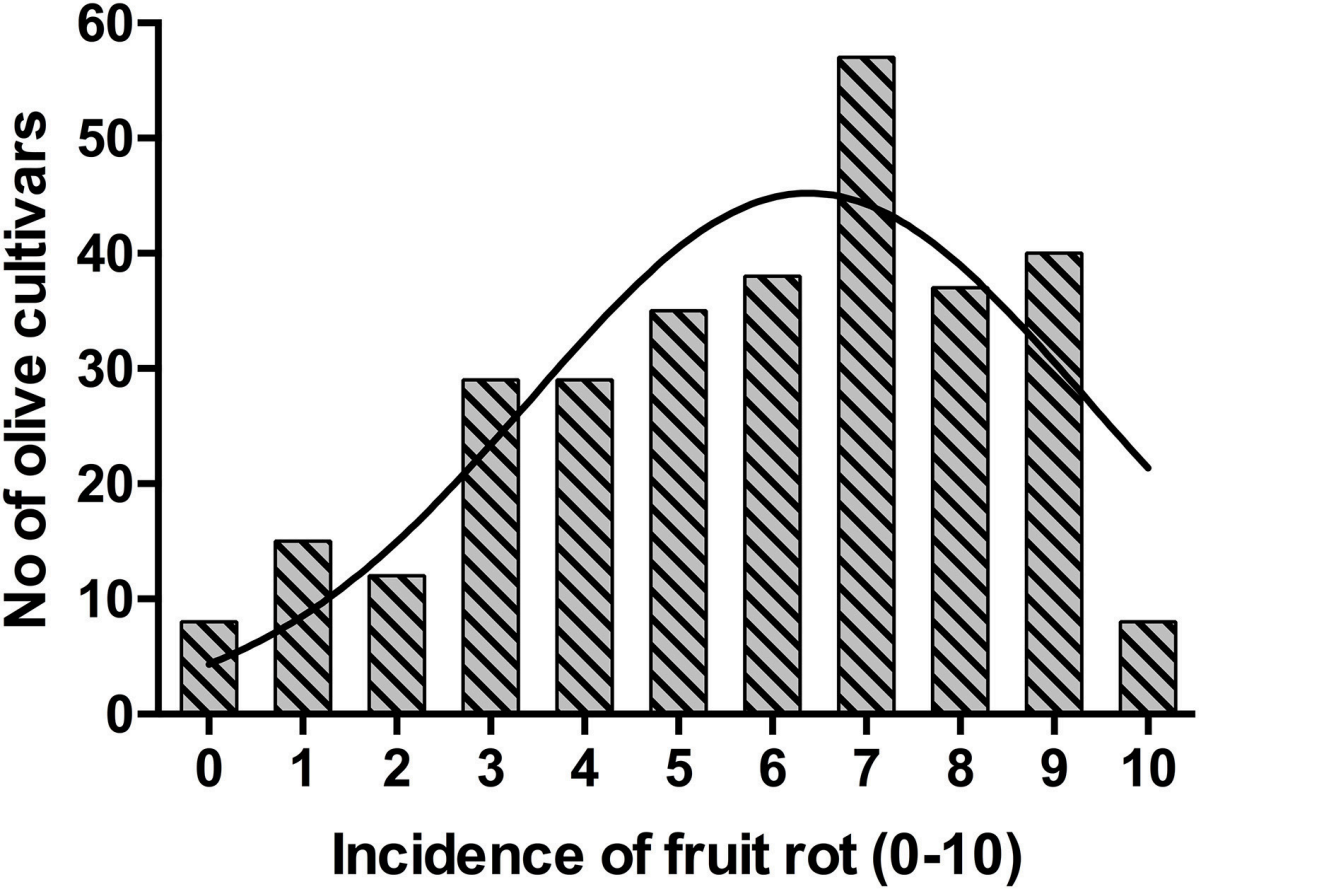
Frequency distribution of fruit-rot incidence caused by *Colletotrichum acutatum* in 308 olive cultivars in the World Olive Germplasm Bank of Córdoba (Spain).

**Figure 3:**
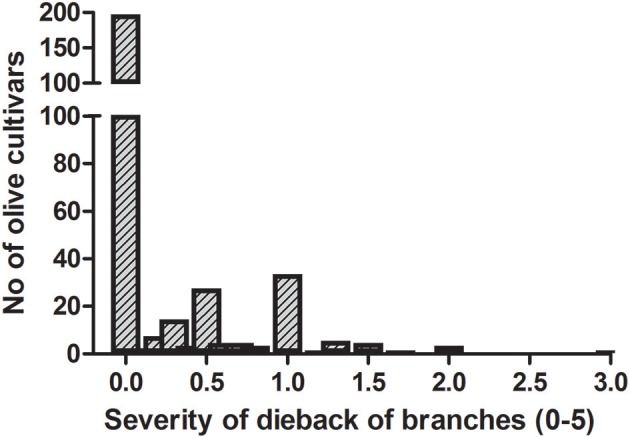
Frequency distribution of branch dieback caused by *Colletotrichum acutatum* in 308 olive cultivars in the World Olive Germplasm Bank of Córdoba (Spain).

**Figure 4:**
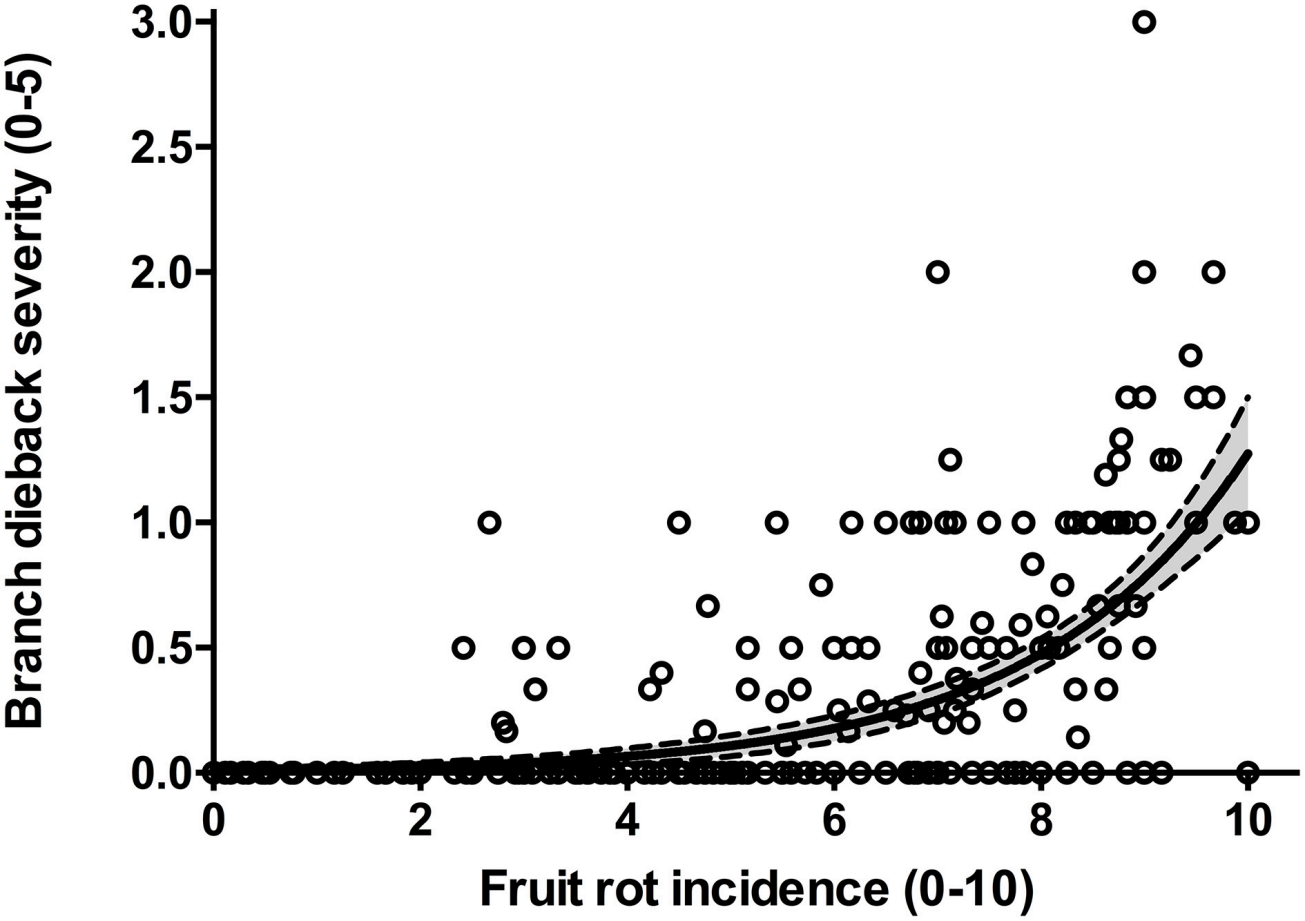
Relationship between fruit-rot incidence and branch-dieback severity caused by *Colletotrichum acutatum* in 308 olive cultivars in the World Olive Germplasm Bank of Córdoba (Spain). Lines represent the fitted exponential growth equation *Y* = 0.009 × e^0.492^ and the confidential intervals at 95%.

The frequency distribution of cultivars according to the categories of fruit-rot incidence is skewed to the left (skew parameter *k* = −0.436) and had a negative kurtosis (*k*_*u*_ = −0.694), which highlighted the prevalence of S or HS cultivars in the collection (Figure 2). Instead, the frequency distribution of cultivars according to the categories of branch-dieback severity showed a positive skew (*k* = 2.01) and a positive kurtosis (*k*_*u*_ = 4.907) due to the fact that most of cultivars did not show this second disease syndrome since it only occurred in some cultivars that had a high fruit-rot incidence (Figure 3). There was also a significant correlation (Spearman rank correlation: *r* = 0.530, *P* < 0.001) between the fruit-rot incidence and the volume (%) of tree canopy affected by the pathogen for all of the cultivars. This relationship was weak albeit it improved when we only considered the cultivars that showed a fruit-rot incidence higher than 2.5 (0.71% of affected fruit). A simple exponential growth curve was well fitted to the values of volume (%) of affected canopy over fruit-rot incidence (Figure 4). Even so, some cultivars that showed high values of fruit-rot incidence did not show branch dieback, such as “Bouteillan,” “Grosal de Cieza,” “Imperial,” “Manzanilla de Almería,” and “Picudo Blanco de Estepa,” which had a fruit-rot incidence >9 but had no dieback symptoms. On the other hand, the cv. Salonenque olive trees showed most of their fruit affected by the pathogen (severity value = 9) and, also, a 50% branch-dieback (severity value = 3) of their canopy affected by dieback of branches.

Comparisons for each country between the frequency of resistant cultivars (HR and R categories) and the frequency of susceptible ones (MS, S, and HS categories) were conducted to determine whether the origin of the cultivars had any effect on their susceptibility. The null hypothesis in these tests was that there was no prevalence of any category of susceptibility while the alternative hypothesis was, that there was a prevalence of resistant or susceptible cultivars. Overall, there was a significant dominance of susceptible cultivar for all of the cultivars. Conversely, when the comparisons were individually conducted among cultivars from the same country, there was no prevalence of resistant or susceptible cultivars in most cases, probably due to the low number of cultivars available. However, the susceptible cultivars were dominant in Spain and Syria while resistant ones were prevalent in Italy (Table 4).

**Table 4:** Origin and susceptibility to anthracnosis of the olive cultivars evaluated in the World Olive Germplasm Bank of Córdoba (Spain).

**ORIGIN**	**Cultivars (No)**	**HR^a^**	**R**	**MS**	**S**	**HS**	***P*-value^b^**	**R/S^c^**
ALBANIA	7	2	2	3	0	0	0.3103	-
ALGERIA	1	0	1	0	0	0	0.2632	-
CHILE	1	0	0	0	0	1	0.4449	-
CROATIA	7	2	1	3	1	0	0.5480	-
CYPRUS	2	1	1	0	0	0	0.1136	-
EGYPT	3	1	1	1	0	0	0.3539	-
FRANCE	7	0	1	2	1	3	0.1693	-
GREECE	16	3	2	4	5	2	0.2752	-
IRAN	4	0	3	1	0	0	0.1758	-
ISRAEL	2	0	0	1	0	1	0.2800	-
ITALY	20	9	5	1	3	2	0.0082	R
LEBANON	2	1	1	0	0	0	0.1136	-
MEXICO	2	0	1	0	1	0	0.6850	-
MOROCCO	1	0	1	0	0	0	0.2632	-
PAKISTAN	1	0	0	1	0	0	0.4449	-
PORTUGAL	6	0	0	3	3	0	0.0613	-
SPAIN	174	9	28	34	55	48	0.0000	S
SYRIA	29	1	5	6	11	6	0.0249	S
TUNISIA	6	2	2	0	0	2	0.1898	-
TURKEY	15	1	6	4	3	1	0.3284	-
USA	2	0	0	2	0	0	0.2800	-
TOTAL	308	32	61	66	83	66	0.0000	S

a *HR = highly resistant (0–2 in the 0-0 rating scale), R = resistant (2.1–4.3), MS = intermediate (4.4–6.2), S = susceptible (6.3–7.9), and HS = highly susceptible (8–10)*.

b *P-value of the chi-square test used to determine the dominance or non-dominance of resistant or susceptible cultivars*.

c *R = dominance of resistant cultivars, S = dominance of susceptible cultivars. - = no dominance of resistant or susceptible cultivars*.

The pathogen *C. acutatum s. lat*. was isolated from sampled leaves, shoots, and branches with dieback symptoms albeit with a relatively low frequency (<2.8%).

### Susceptibility of cultivars in artificial inoculation

**Figure 5:**
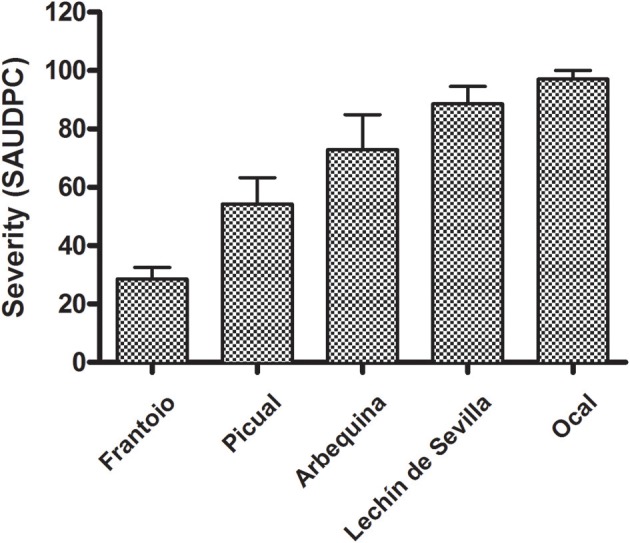
Standard Area Under Disease Progress Curve (SAUDPC) of five reference olive cultivars inoculated with *Colletotrichum acutatum*. All the cultivars were significantly different according to the Fisher's protected least significant difference (LSD) test at *P* = 0.05.

All inoculated cultivars developed typical anthracnose (soapy-rot) with significant differences among them. None of the non-inoculated fruit showed disease symptoms during the 80 days of incubation in humid chambers. The first symptoms were observed in the fruit of the cv. Ocal at 5 days after inoculation, whereas the fruit of the cv. Frantoio showed the first symptoms 23 days after inoculation. Likewise, the pathogen caused the complete rot (severity = 5) of all the fruit of the cvs. Lechín de Sevilla and Ocal on 21 days, while it needed more than 80 days to cause the completely rot of all of the fruit of the cv. Frantoio. In this latter cultivar, only the 17% of inoculated fruit had been shown anthracnose symptoms 40 days after inoculation. The SAUDPC analysis significantly separated the five inoculated cultivars (Figure 5). Finally, there was a significant correlation (*r* = 0.989, *P* = 0.0015) between the SAUDPC of inoculated fruit and the fruit-rot incidence in the field.

## Discussion

Here, we present the largest evaluation of olive cultivars for their resistance to anthracnose, which is considered the most important fruit disease of this crop (Cacciola et al., 2012; Moral et al., 2014). We evaluated the reaction of 308 well-identified cultivars growing in the WOGBC during three epidemic seasons. Among others, two important reasons for which the WOGBC is an excellent experimental plot to evaluate the resistance to *C. acutatum s. lat*. are: (i) the plot is located in an endemic area for anthracnose due to the proximity of the biggest river of Andalusia (Guadalquivir river); (ii) the plot has a high number of HS olive cultivars that act as an inoculum source of *Colletotrichum* (Moral et al., 2015). In a previous evaluation, we presented a limited number of olive cultivars based on a single observation (Moral et al., 2005) and without the correct identification of the trees using molecular markers (Trujillo et al., 2013). In the latter study, Trujillo et al. (2013) clarified numerous problems of homonymy, synonymy, and wrong denominations in the WOGBC. For example, the original cultivars Arauco, Cañivano Blanco, Carrasqueño de Lucena, and Razzola, which were planted in the WOGBC, have been identified as synonymous of the cultivars Azapa, Picholine Marocaine, Picual, and Frantoio, respectively (Trujillo et al., 2013).

Olive fruit infection occurs at all stages of its development, from flower bud emergence to ripening (Moral et al., 2009). These infections occur mainly as a result of water-splashed conidia. Also, the anthracnose disease cycle can be influenced by the activity of olive fruit fly (*B. oleae*), which may increase the fruit's susceptibility by causing wounds or directly act as a spores carrier (Malacrinò et al., 2015). Fruit ripeness increases fruit susceptibility to anthracnose (Moral et al., 2008), with the unripen (or developing) fruit being very resistant to the pathogen, regardless of cultivar (Moral et al., 2008; Cacciola et al., 2012; Moral and Trapero, 2012). Therefore, cultivar resistance is evaluated by inoculating yellowish-green fruit with a spore suspension of the pathogen (Moral et al., 2008; Talhinhas et al., 2015). In any case, the use of this rating scale under field conditions to evaluate the cultivar reaction to the pathogen provides an important (>20-fold) economic saving respect to the artificial inoculation method (Moral, 2009). However, the correct evaluation of the olive cultivars under field conditions can only be conducted during epidemic seasons (Moral and Trapero, 2009), which are sporadic in Mediterranean conditions (Cacciola et al., 2012; Moral and Trapero, 2012). In our case, there were only three epidemic seasons during a period of 10 years. The epidemic intensity during the studied years (Figure 1) was associated with conducive weather conditions for anthracnose, mainly the annual rainfall. Thereafter, the greatest epidemic was in 1997, a wet year (888.4 mm) in Cordoba, which had been preceded by a very wet 1996 (951.1 mm). Furthermore, the epidemic years 2005 and 2006 were moderately wet with an annual rainfall of 548.8 and 560.7 mm, respectively.

In this study, the phenotypic resistance of the cultivars was very stable, as shown by the fact that the experimental error explained only around 10% of the total variance (η^2^), while the cultivar genotype explained over a 65% of this. This fact implies a high stability of the cultivar response to the pathogen (resistance/susceptibility) during the epidemic years. The season (which includes its weather conditions) and the interaction cultivar-season had also a similar effect as that of the experimental error. We did not conduct anthracnose evaluations during non-epidemic seasons due to the almost complete absence of the disease during these years, even in the HS cultivars (Moral, 2009). In other words, anthracnose is a highly weather-dependent disease (Moral and Trapero, 2012).

As it was previously described, the cultivar reaction to *Colletotrichum* is a continuous variable ranging from HR to HS cultivars (Moral et al., 2015). Nevertheless, the susceptibility/resistance of the cultivars is more useful and easily understood by the farmers and agronomist if the cultivars are placed into distinct ordinal classes (Pataky et al., 2011). In this study, as in previous studies of olive diseases (Moral et al., 2005; Trapero and López-Doncel, 2005), we classified the cultivars in five categorical groups using a K-means analysis that minimized the variances. The intervals (the ranging values) of each categorical group can shift slightly depending on the number of evaluated cultivars since K-analysis uses a centroid value (average of the data of each group) for each given categorical group (Jain et al., 1999).

In general, susceptible cultivars (MS, S, and HS, total 215 cultivars) were more prevalent than resistant cultivars (R and HR, total 93 cultivars) regardless of country of origin, except for Italy. For Italian cultivars, resistance to the pathogen (*C. godetiae*) in the WOGB was prevalent. Since *Colletotrichum* spp. is endemic in north of Italy, most of these Italian cultivars could be selected by the farmers according their resistance to the pathogen (Barranco et al., 2000; Bartolini and Cerreti, 2017). The studied Italian cultivars also show a high degree of genetic homogeneity, for example, they all belong to the chlorotype group E1.1, except the S cultivar Carolea that belongs to the group E1.2 (Besnard et al., 2013). Likewise, all the Italian cultivars in the WOGBC, which belong to the genetic cluster 2 described by Trujillo et al. (2013), are resistant to anthracnose except “Cipressino.”

In the extremes of the resistance/susceptibility, we found the cultivar Dolce Agogia, which showed a complete resistance to the pathogen (i.e., absence of disease symptoms), and the cultivars Acebuchera, Picudo Blanco de Estepa, and Uovo Piccione, in which the fruit-rot incidence was 100%. In this study, we described most of the evaluated cultivars as susceptible (MS, S, or HS) to the pathogen, including the species *Olea ferruginea*, which was moderately susceptible. Results of present study agree with previous observations for susceptible cultivars: Ascolana tenera, Barnea, Galega vulgar, Gordal sevillana, Hojiblanca, Manzanilla de Sevilla, Morisca, Ocal, Picudo, Sant Agostino, and Verdial de Badajoz; and for the resistant or moderately resistant cultivars: Bical de Castelo Branco, Coratina, Frantoio, Empeltre, Leccino, Manzanilla cacereña, Mixani, and Picual (Barranco et al., 2000; Rallo et al., 2005; Moral et al., 2015; Talhinhas et al., 2015; Bartolini and Cerreti, 2017). Conversely, the published response to anthracnose of some cultivars does not match with our current results. In our study, four of these cultivars [“Abou-Salt Mohazan,” “Azapa” (syn. “Arauco”), “Blanqueta,” and “Cordovil de Castelo Branco”] were more susceptible and seven of them (“Arbequina,” “Cobrançosa,” “Itrana,” “Moraiolo,” “Morrut,” and “Picholine”) were somewhat less susceptible than their respective previous classification (Bartolini and Cerreti, 2017).

Errors in the classification of olive cultivars according to their resistance to anthracnose have been extensively discussed by us and they are usually associated with: cultivar misidentification, effect of the ripeness time during the evaluation moment, low inoculum pressure, unfavorable environmental conditions for disease development, and confusion with other fruit-rot diseases caused by species of *Alternaria, Botryosphaeria, Fusarium*, or *Neofabraea* (Moral et al., 2008, 2014, 2015; Moral and Trapero, 2009). Furthermore, the potential interaction between *Colletotrichum* species (or isolate) and the olive cultivar needs a special mention. In general, this type of interaction has been described with MS and S cultivars, such as “Galega vulgar,” “Cobrançosa,” or “Hojiblanca.” Fortunately, R or HR cultivars, such as “Blanqueta,” “Picual,” or “Frantoio” show a high degree of resistance to the different isolates (Xaviér, 2009; Talhinhas et al., 2015). Concomitantly, some *Colletotrichum* species (or isolates) tend to be weakly (e.g., *C. acutatum s. str*. or *C. rhombiforme*) or highly virulent (e. g. *C. godetiae* or *C. nymphaeae*) against a broad range of olive cultivars (Xaviér, 2009; Schena et al., 2014; Talhinhas et al., 2015).

The histogram of resistance/susceptibility of the olive cultivars according to fruit-rot incidence showed that data fit a normal distribution but with a dominance of the susceptible cultivars. Similarly, a substantial deviation from normal distribution among cultivars for different agronomic characteristics has been described, such as yield, ripening data, and oil content (Caballero et al., 2006; León et al., 2008, 2011). Although our study was not intended to address the resistance mechanisms against the pathogen, it suggests a complex and polygenic control of the resistance to *Colletotrichum* species. In any case, other types of genetic control, including combinations of minor and major genes, could be involved in the resistance of the olive tree to *Colletotrichum* species (Geffroy et al., 2000). However, the case of major genes mediating the defense against non-biotrophic pathogens is rare (Poland et al., 2009). In addition, phenolic compounds have an important role in the defense of the fruit against fungal pathogen on different crops (Prusky, 1996), including olive (Moral et al., 2015); in this case, and due to phenolic acids derivate from different metabolic pathways in the plant (Boudet, 2007), there is not a clear relationship between genes and phenolic compound. Fortunately for the olive breeding programs, crosses between resistant cultivars produce a high frequency of resistant descendants (Moral et al., 2015).

Likewise, important differences have been observed according to the severity of branch-dieback among olive cultivars. This second syndrome of olive anthracnose is associated with the production of phytotoxins by the pathogen on rotten fruit (Moral et al., 2009, 2014). In our study, both fruit-rot incidence and branch-dieback severity appear correlated albeit some of the cultivars, which showed high values of fruit-rot incidence, did not show dieback symptoms. These results suggest differences in the resistance mechanisms for both syndromes. In the experimental plot, the percentage of isolation of *Colletotrichum* in semi-selective medium from symptomatic tissues of the olive trees were relatively low (<3%) during spring-summer and it was associated with the rainfall events. Schena et al. (2017), using a duplex qPCR, have described a high colonization of these vegetative tissues from May to October. In our conditions, we have never observed fruiting bodies (acervuli) of the pathogen on leaves or shoots under field conditions (Moral et al., 2009), although they can be induced in humid chamber after 1 month of incubation (Moral et al., 2014). For this reason, we think that these tissues have a limited role as inoculum sources in comparison with affected fruit in southern Spain (Moral and Trapero, 2012; Moral et al., 2014). In Southern Italy, the pathogen is able to infect directly leaves and shoots beside, these latter tissues can be also colonized by the pathogen through the peduncles of rotten fruit (Martelli, 1960). Furthermore, acervuli of the pathogen have been described on olive leaves in other countries, such as Australia and Italy (Martelli, 1961; Sergeeva et al., 2008). These differences could be due to variation among pathogen populations, weather conditions, or cultivar resistance.

It is worthy of note that the evaluation of the severity of branch-dieback caused by *Colletotrichum* sp. may mislead the inexperienced evaluator since other pathogens can cause similar symptoms. In the event of doubt, the evaluator should conduct isolation for other candidate pathogens. For example, we diagnosed Verticillium wilt in more than 20 olive trees belonging to different cultivars in the WOGBC (Morello et al., 2016).

Current trends in planting high-density orchards (which are very conducive for olive anthracnose) and reducing the use of copper-based fungicides are contrary to the high-quality oils demanded by consumers (Moral et al., 2012, 2014; Díez et al., 2016). Thereafter, the selection of less susceptible cultivars to anthracnose is essential for new plantations. The information about the resistance to olive anthracnose that is presented in this study is fundamental for farmers, technicians, and breeders.

## Conclusions

In this paper, we evaluated the resistance of 308 olive cultivars to *C. acutatum s. lat*. during three epidemic seasons under field conditions. However, there is a clear predominance (69.7%) of susceptible cultivars (MS, S, and HS), we have also identified 32 cultivars (10.4%) highly resistant to the pathogen. The most notable cultivar was “Dolce Agogia,” which did not show any anthracnose symptom during the three seasons. This work constitutes the largest evaluation of olive cultivars according to their resistant to *C. acutatum* to date, albeit the response of other many cultivars to the pathogen is not well-known and, thus, it should be evaluated. For future work, the methodology described in this paper should be also used to evaluate the cultivar response to other aerial fungal pathogen affecting leaves and fruit, such as *V. oleaginea* and *Pseudocercospora cladosporioides*, causal agents of peacock spot and cercosporiosis, respectively.

## Author contributions

Conceived and designed the study: AT and JM. Performed the evaluation under field conditions: JM, JRV, CX, and LFR. Curator of the WOGBC: JC. Performed the evaluations in controlled conditions: JM. Analyzed the data: JM and AT. Wrote the paper: JM and AT.

### Conflict of interest statement

The authors declare that the research was conducted in the absence of any commercial or financial relationships that could be construed as a potential conflict of interest.

## Footnotes

^1^http://www.ine.es.
